# Enhancement of Family-Centred Care Is Associated with a Reduction in Postmenstrual Age at Discharge in Preterm Infants

**DOI:** 10.3390/children11111316

**Published:** 2024-10-29

**Authors:** Rahel Schuler, Carola Eiben, Markus Waitz, Bernd A. Neubauer, Andreas Hahn, Walter A. Mihatsch

**Affiliations:** 1 Department of General Pediatrics and Neonatology, Justus-Liebig-University, Feulgenstrasse 12, D-35392 Giessen, Germany; carola.eiben@paediat.med.uni-giessen.de (C.E.); markus.waitz@gnh.net (M.W.); 2 Department of Pediatric Neurology, Justus-Liebig-University, Feulgenstrasse 12, D-35392 Giessen, Germany; bernd.a.neubauer@paediat.med.uni-giessen.de (B.A.N.); andreas.hahn@paediat.med.uni-giessen.de (A.H.); 3 Division of Neonatology and Pediatric Intensive Care Medicine, Department of Pediatrics and Adolescent Medicine, University Medical Center Ulm, Eythstr. 24, D-89075 Ulm, Germany; walter.mihatsch@uni-ulm.de; 4 Department of Health Management, Neu-Ulm University of Applied Sciences, D-89231 Neu-Ulm, Germany

**Keywords:** early discharge, family-centred care, parental satisfaction, home tube feeding, home caffeine therapy, home monitoring

## Abstract

Background/Objectives: Long hospitalisation has been recognized as an independent risk factor for poor neurodevelopmental outcomes of preterm infants. Systematic training and early inclusion of parents in their preterm infant’s care is a strategy to shorten the length of hospital stay. We implemented an enhanced stepwise family-centred care program and assessed its effects on postmenstrual age (PMA) at discharge and parental satisfaction. Methods: This prospective single-centre longitudinal cohort study was carried out in a German level III neonatal unit from October 2020 to May 2023. Five consecutive 6-month cohorts (1 baseline and 4 intervention cohorts, 169 infants and their caregivers) were analysed. Results: Mean PMA at discharge did not change in the total cohort but declined significantly in patients without neonatal morbidities from baseline to cohort 4 (37.2 ± 1.4 to 36.1 ± 1.6 weeks; *p* = 0.036). Concomitantly, discharge with tube feeding raised from 2.4% to 74.1% (*p* < 0.001) and discharge with home monitoring raised from 9.8% to 74.1% (*p* < 0.001), while unplanned readmissions remained unchanged (*p* = 0.44). Parental satisfaction with time point of discharge increased non-significantly from baseline to cohort 4 (75.8% vs. 95.7%; Chi^2^ 0.22). Conclusions: Discharge of preterm infants at a significantly lower PMA is feasible through enhancement of family-centred care and is very well accepted by parents.

## 1. Introduction

Length of hospital stay was identified as an independent risk factor associated with poor neurodevelopmental outcome of preterm infants at one to two years [1]. Neonatal intensive care unit (NICU) surroundings, such as light, noise, and pain, as well as separation from the parents, were recognised as risk factors associated with neurodevelopmental impairment [1,2]. As length of stay and postmenstrual age (PMA) at discharge increase with decreasing PMA at birth, the most immature infants are exposed the longest to these unfavourable surroundings [3,4]. Acknowledging the negative impact of prolonged hospital stay, Lundberg et al. suggested that discharge should be as early as medically safe [5]. Currently, there are no recommendations on optimum length of stay for preterm infants [6,7,8], and there is considerable variability regarding criteria for discharge at the national [4,6,9] and international levels [7,10], which cannot be solely explained by biological factors [11]. Instead, these variations seem to depend on differing local practices and guidelines [12]. Although it has been found that safe early discharge is principally possible and well accepted by parents [5,13], in our unit preterm, infants of ≤32 + 0 weeks and/or birthweight ≤1500 g were discharged at approximately 38 weeks PMA. Besides physiological stability of the infant, empowerment of parents and extension of care to the family home after discharge seem to be crucial factors enabling an early discharge [5,11].

The aim of the present study was to assess whether implementation of parental and staff education alongside empowerment of parents through family-centred care (FCC) can facilitate discharge at an earlier PMA in our unit, and if so, whether this is true for preterm infants with and without neonatal morbidities. To accomplish this, we analysed the effects of a stepwise enhancement of FCC in four consecutive 6-month cohorts of preterm infants born between June 2021 and May 2023. Parental satisfaction and preparedness with early discharge were systematically analysed by questionnaire. Unplanned hospital readmission was assessed as a marker of medical safety [5].

## 2. Materials and Methods

### 2.1. Study Design

This study included preterm infants of a PMA of ≤32 + 0 weeks and/or birthweight ≤ 1500 g and is part of a prospective longitudinal study assessing the impact of stepwise enhancement of FCC in a German perinatal level III centre on infant, parent, and staff outcomes. In Germany, a level III perinatal centre is the highest level, treating preterm infants from 22 + 0 weeks PMA and surgical preterm and term infants. The project is registered at clinicaltrials.gov (NCT05286983, 18 March 2022) and was approved by the local Institutional Review Board prior to study start (AZ 153/20) [14]. The primary outcome of the study is PMA at discharge. Secondary outcomes are PMA at discharge in infants without neonatal morbidities, parental satisfaction with the time point of discharge, parental stress with feeding at home, and parental preparedness for discharge across five study cohorts.

Subjects were eligible if they met the following inclusion criteria: inborn or outborn preterm infants of a PMA of ≤32 + 0 weeks and/or birthweight ≤ 1500 g. Infants were ineligible if they had severe congenital anomalies (e.g., cyanotic heart disease, severe lung hypoplasia, …), if decision for palliative care was taken before study entry, or if parents suffered from a severe active psychiatric disease (e.g., psychosis). Written informed consent was obtained from both caregivers.

While the stepwise intervention was applied to all infants admitted to the neonatal unit, only those preterm infants meeting inclusion criteria and not meeting exclusion criteria were eligible for the study.

Neonatal morbidities were defined as follows: Intraventricular haemorrhage (IVH) > grade III and PVL as diagnosed by ultrasound [15], retinopathy of prematurity (ROP) > stage 3 or treatment of ROP, necrotising enterocolitis (NEC) > Stage 2 [16], and bronchopulmonary dysplasia (BPD). BPD was defined according to the physiological definition by Walsh [17]. At 36 weeks PMA, infants on mechanical ventilation, continuous positive airway pressure (CPAP), or oxygen > 0.3 were diagnosed with BPD. The infants with oxygen < 0.3 underwent a defined stepwise oxygen reduction test, and if failed, they were also diagnosed with BPD [17].

### 2.2. Intervention

The baseline cohort consisted of 45 preterm infants and their parents prior to the introduction of FCC elements admitted from October 2020 to May 2021. Forty-five infants were the average number of patient admissions per six months fulfilling the inclusion criteria during the previous five years. At this time, it was our policy to discharge infants after reaching a PMA of at least 35.0 weeks, and generally without tube feeding or home monitoring. A few days before discharge, rooming in with one parent (mostly the mother) was initiated. A socio-medical follow-up program after discharge had been in place for several years prior to study start. A nurse or a social worker visited the family at least once a week for 12 weeks after discharge to monitor weight gain, answer questions, and assist with medical appointments. During the baseline cohort, parents could only be present in the unit for 5–6.5 h per day.

The remaining cohorts consisted of infants and their parents recruited consecutively within 6-month periods: cohort 1 (June–November 2021), cohort 2 (December 2021–May 2022), cohort 3 (June–November 2022), and cohort 4 (December 2022–May 2023). Starting in June 2021, new FCC elements were introduced. This process was led by a team of physicians and nurses with a special interest in FCC.

In cohort 1, the main change from the baseline cohort was the liberalisation of access hours to 22.5 h in the NICU and to 24 h per day in the stepdown unit. Biweekly educational staff workshops were initiated. The importance of parental presence and the positive effects of parental involvement as the primary caregiver of their infant were discussed.

In cohort 2, parents were actively encouraged but not obliged to spend at least 6 h with their infant, since liberalisation of access had led to minor improvements in presence lengths only [18]. Parental education on being the primary caregiver of their infant and increasing active involvement was conducted individually at the bedside. To monitor and motivate parental progress in caregiving skills, a weekly self-assessment questionnaire was introduced [19]. The questionnaire assessed 22 caregiving skills including diaper change, tube feeding, and bottle or breast feeding (Supplement S1). Mothers and fathers assessed separately whether they or the nurse performed specific care activities and were encouraged to gradually take over more activities. Additionally, the parent lounge was renovated and more space for parents was created.

In cohort 3, a weekly parent support group was initiated, the discharge process was improved, and discharge became possible at the PMA of 34.0 weeks with home monitoring. The parent support group included sessions on self-care, benefits of parental presence, breastfeeding, early discharge, and follow-up after discharge. As parents often felt unprepared and surprised if informed about their discharge within a few days [20], parents were educated early on during the NICU stay that discharge usually will take place at a PMA of 35–36 weeks, likely with tube feeding and possibly home monitoring. Additionally, a checklist of tasks to be accomplished (Supplement S2) was developed, regularly reviewed, and gradually completed with the parents starting at a PMA of 32.0 weeks.

In cohort 4, medical rounds were implemented once a week together with the actively involved parents, who were excluded before.

### 2.3. Outcome Parameters

PMA at discharge was used as the primary outcome parameter. Secondary outcomes were PMA at discharge in infants with and without neonatal morbidities, and mean PMA at discharge in infants without neonatal morbidities adjusted for the PMA at birth and birthweight. Further secondary outcomes were tube feeding at discharge, home monitoring and oral caffeine therapy at discharge, planned and unplanned readmissions within 4 weeks after discharge, parental satisfaction with the discharge time point, parental stress level with feeding at home, and parental preparedness for discharge. For achieving comparability with the literature, a post hoc analysis was performed including only very preterm infants [10].

### 2.4. Data Collection

Patient characteristics and readmissions within 4 weeks after discharge were extracted from the medical records. Parents’ opinions on whether the timing of discharge was appropriate (“too early”, “right”, “too late”), their stress level with feeding at home, and their preparedness for discharge were determined based on a previously published self-assessment questionnaire 4–6 weeks after discharge [18]. Stress level and preparedness for discharge were assessed using 5-point Likert-type scales with 5 being very stressed and 5 very well prepared. This questionnaire was either handed out during routine follow-up visits or sent via mail. If the questionnaire was not returned, parents were contacted via telephone. For non-German-speaking parents, the questionnaires were completed with a certified translator.

### 2.5. Statistical Analysis

Data analysis was conducted using SPSS version 28.9.1.1. (14) (IBM Corp., Armonk, New York, NY, USA). Data were presented as the mean (standard deviation, SD) or as number (percentage) as appropriate. The chi-square test was used to compare categorical variables between the five cohorts ANOVA, together with a post hoc Dunnett test comparing cohort 4 with the baseline cohort, was used to compare continuous variables between the groups. ANCOVA was used to compare the mean PMA at discharge in infants without neonatal morbidities adjusted for the PMA at birth and birthweight. A *p*-value < 0.05 was considered statistically significant.

## 3. Results

Of 228 principally eligible infants, a total of 169 infants were included in this study. Ten parents declined consent, 30 fulfilled exclusion criteria, and 19 were not approached due to staffing shortage. In addition, three infants (PMA 23.7 weeks, PMA 24 weeks, PMA 27.7 weeks) in the baseline cohort died on the 26th, eighth, and sixth day of life and were excluded from further analysis (Supplement S3). Infants had a PMA from 22.0 to 34.0 weeks PMA.

Clinical characteristics, complications during hospital stay, characteristics at discharge, and readmission of all 166 surviving infants in the five cohorts are summarised in Table 1, Table 2 and Table 3. Significant differences between the single cohorts were found for tube feeding at discharge, home monitoring, and oral caffeine therapy at discharge, as well as planned readmission, which were all significantly more frequent in cohort 4. The frequency of neonatal morbidities such as BPD, IVH ≥ grade III, PVL, ROP ≥ stage 3 or treatment of ROP, and NEC ≥ stage 2 differed not significantly between the single cohorts. Overall, 24 infants (14.5%) developed at least one neonatal complication.

The mean PMA at discharge across all cohorts independent of the presence of neonatal morbidities declined non-significantly from 37.8 weeks (±2.1) in the baseline cohort to 36.4 weeks (±1.9) in cohort 4 (*p* = 0.21).

**Figure 1:**
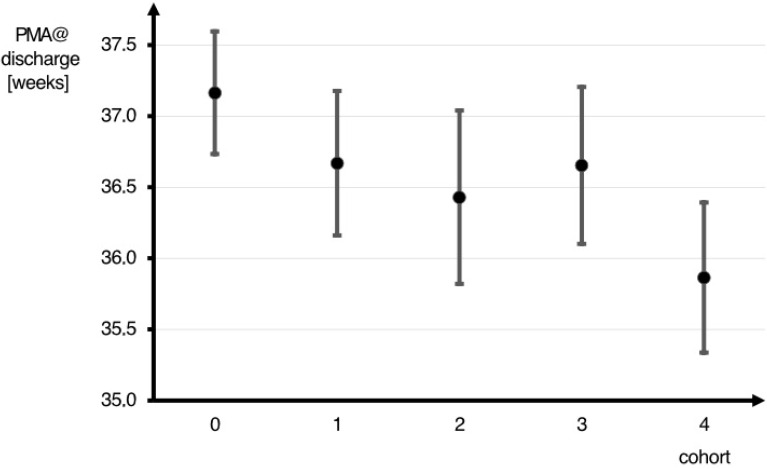
Postmenstrual age at discharge of infants without neonatal morbidities, corrected for gestational age and birthweight, decreased significantly (*p* < 0.01).

Table 4 depicts the data for infants without neonatal morbidities alone. There was a significant decline in the PMA at discharge from baseline to cohort 4 (37.2 (±1.4) to 36.1 (±1.6) weeks (*p* = 0.036)) (Table 4). This effect was robust and remained significant (*p* < 0.01) after adjusting for the PMA age at birth and birthweight (Figure 1). Discharge with tube feeding significantly increased from 2.4% (1/41) in the baseline cohort to 74.1% (20/27) in cohort 4 (*p* < 0.001). Similarly, significantly more infants in cohort 4 (74.1% = 20/27) were discharged with home monitoring compared to the baseline cohort (9.8% = 4/41) (*p* < 0.001). No infant was discharged with oral caffeine therapy in the baseline cohort compared to 40.7% (11/27) in cohort 4 (*p* < 0.001). Unplanned readmissions of infants without neonatal morbidities did not differ significantly between the cohorts (*p* = 0.44). Planned readmissions of infants without neonatal morbidities increased significantly from the baseline cohort to cohort 4 (*p* = 0.008).

Table 5 shows the parental assessment of the appropriateness of discharge of infants without neonatal morbidities. 84.5% of questionnaires were retrieved. Most parents assessed the time point of discharge as appropriate across all cohorts. (*p* = 0.15). The preparedness for discharge was rated as high (4–5/5) by most parents across all cohorts (*p* = 0.19). High stress level (4–5/5) with feeding was reported by 9.4% of the parents in the baseline cohort and 27.3% to 33.3% in cohorts 1–4. (*p* = 0.31).

## 4. Discussion

Empowerment of parents by systematic enhancement of FCC led to a non-significant decline of the PMA at discharge in all infants. Most of the study population (85%) was discharged without neonatal morbidities such as BPD, severe IVH, severe ROP, or NEC. In this subgroup, the PMA at discharge declined significantly by approximately one week. This suggests that discharge of patients with complications might be delayed by medical and organisational challenges such as availability of homecare nursing. Earlier discharge appeared to be safe, as unplanned readmissions did not increase.

Parents very much appreciated the concept of earlier discharge. In the two final cohorts, virtually all parents (>97%) felt that timing of discharge was appropriate in contrast to only approximately three quarters in the baseline cohort. Notably, only one family judged the time point of discharge as too early. In contrast to the first cohorts, no parent assessed the time point of discharge as too late. About 90% of parents felt well or very well prepared for infant care at home. Of interest, about one third of parents experienced high stress at home with feeding throughout the study. Therefore, our future FCC program needs to address this issue and must support parental resilience towards infant feeding difficulties.

In early discharge programs in Sweden, Finland, and Denmark, preterm infants of a PMA of 32.0–33.6 weeks could be discharged at a PMA of 35.6–35.9 weeks [5,21,22]. Although the PMA at discharge has been described to increase with decreasing PMA [3], we were able to discharge much more immature infants in cohort 4 (PMA 28.1 ± 2.9 weeks) at an only marginally higher PMA (36.4 ± 1.9 weeks) than in the Scandinavian studies. Similar to our baseline cohort (PMA at discharge 38.7 weeks), a recent study from six European neonatal networks including preterm infants with a PMA of 24.0–29.9 weeks reported a discharge at a mean PMA of 37.9–38.9 weeks [10]. By enhancement of FCC, we were able to discharge this immature subgroup of preterm infants approximately 2 weeks earlier (mean PMA of 36.4 weeks, post hoc analysis, Table 2). After having spent several weeks in hospital a 2-week reduction in average length of stay is very relevant for the families as the hospital situation is often perceived as stressful and burdensome [23]. Moreover, earlier discharge also increases admission capacity and reduces hospital costs [24].

Acknowledging parents’ wishes for early discharge and accepting parents to be capable of caring for more immature infants at home when still dependent on medical support was a change in our unit’s mindset. Our approach was a stepwise introduction of new FCC elements starting with organisational changes in the first cohort, such as liberalisation of access hours and regular staff education. The focus in the following cohorts was on increasing parental involvement as the primary caregiver of their infant. Tube feeding, for example, was newly redefined as routine parental care competence and was taught early on during the NICU stay. Transition to home remains a critical phase for parents [25], and despite the long hospital stay, parents of preterm infants often feel unprepared for discharge and for caring independently for their infant [20,26]. Acknowledging this period as a vulnerable phase, parents were involved from a PMA of 32 weeks on to actively participate in discharge planning and preparation (Supplement S2). Also, rooming in with one parent was initiated at an earlier PMA and continued for approximately 7–10 days. To improve parental medical knowledge and to provide peer support, the parent support group was initiated, which parents themselves described to be important [27]. Family rounds at the infants’ beds were initiated as well, as this practice was appreciated by parents and improved communication [28]. As also reported by other authors, empowering parents by gradual enhancement of FCC made earlier discharge possible [21,24,29].

Discharge with tube feeding is an important practice to facilitate early discharge [12,30] and was found to be safe, as well as associated with longer breastfeeding and no increase in parental anxiety [31,32,33]. As feedback from parents and the homecare team in the first cohort were very positive, we thought of strategies to make early discharge possible for more families. Apnoea and bradycardia events hindered early discharge, as they often only resolved after term gestation in infants delivered at a PMA of 24–28 weeks [34]. We therefore started to offer home monitoring and oral caffeine therapy at home routinely. Discharge of preterm infants with home monitoring has been described previously [5,12,35] with a wide variation between different sites [36]. Oral caffeine therapy at home was reported to be feasible and safe for stable preterm infants at a PMA of 38 weeks in a single centre [37]. Routine discharge of preterm infants before a PMA of 35 weeks with caffeine was reported by only three of 83 units in a Scandinavian survey without mentioning outcomes or complications [12]. Preterm infants in our study were discharged with oral caffeine therapy at a PMA of 36.5 ± 1.8 weeks without apnoea-related readmissions, suggesting this approach to be safe.

Some infants who passed the oxygen reduction test and therefore formally did not have the diagnosis of BPD, according to Walch [17], failed to stay completely without oxygen and were therefore discharged with home oxygen.

Since there was no increase in unplanned readmissions, early discharge appears to be medically safe. Our unplanned readmission rate of 10% is similar to the rate of 8% observed in another German cohort of very low birthweight infants. [24] The authors also found a trend of declining unplanned and increasing planned readmissions after introduction of an FCC program [24]. In our study, the high planned readmission rate in cohorts 2–4 was due to scheduled surgery (e.g., inguinal hernia), vaccination, or ROP treatment, which occurred during the initial hospital stay in the baseline cohort and cohort 1. In general, the duration of planned readmissions was 48 h.

Although discharge occurred at a lower PMA, with more medical equipment and a high percentage of planned readmissions, the time point of discharge was assessed as appropriate by nearly all parents. This shows that parents highly value being at home with the family and are willing to accept medical equipment. Therefore, it was our goal to empower parents to feel competent and confident at home at an earlier time point. Our focus shifted from discharge with as little medical equipment as possible to discharge as early as medically safe regardless of necessary equipment.

Throughout this study, parents felt very well prepared for discharge. Although more preparation was necessary in cohort 2–4 at an earlier PMA, this could be successfully achieved through FCC, including earlier engagement of parents and improved discharge planning. The positive attitude of parents towards early discharge is in line with other early discharge programs [11,21,35].

Our study has limitations. As this was a single-centre study, our results cannot easily be transferred to other units. Moreover, this was not a randomised trial, and we cannot exclude confounding factors over time.

However, our results show that parents are capable and positive towards early discharge. Parents can be entrusted with the care for their preterm infant at home at an earlier PMA. Requirement of medical equipment and medication should no longer hinder discharge. Principally, routine discharge of preterm infants with caffeine therapy and home monitoring at a PMA of 33.0 weeks could be feasible. However, this must be accompanied by a continuous scientific assessment of safety and parental satisfaction to avoid overburdening parents.

Future multicentre studies evaluating early discharge based on family-centred care could prove the generalisability of our results. Different models of follow-up (homecare nursing, telemedicine, outpatient department) should be evaluated and compared in future studies on early discharge, as in some areas or countries home-based follow-up may not be available.

## 5. Conclusions

Parents are in favour of early discharge and are willing to accept medical equipment at home to make this possible. Empowerment and training of parents embedded in the FCC program of our perinatal level III centre facilitated discharge of preterm infants without neonatal morbidities at a significantly lower PMA.

## Figures and Tables

**Table 1:** Infant characteristics and neonatal morbidities * of all cohorts.

	Baseline Cohort	Cohort 1	Cohort 2	Cohort 3	Cohort 4	ANOVA *p* Value
*n*	51	35	26	27	30	
PMA at birth, weeks (SD)	28.3 (±3.0)	29.2 (±2.7)	27.9 (±2.8)	29.3 (±2.8)	28.1 (±2.9)	0.33
Infants < 28 weeks PMA, *n*	24	15	17	10	13	
In hospital death, *n*	3 ^a^	0	0	0	0	
Discharged alive, *n*	48	35	26	27	30	
Caesarean section *n* (%)	43 (89.6%)	35 (100.0%)	26 (100.0%)	26 (96.3%)	29 (96.7%)	0.28
Male sex, *n* (%)	18 (37.5%)	18 (51.4%)	15 (57.7%)	12 (44.4%)	16 (53.3%)	0.44
PMA at birth, weeks (SD)	28.5 (±2.9)	29.2 (±2.7)	27.9 (±2.8)	29.3 (±2.8)	28.1 (±2.9)	0.33
Birthweight, g (SD)	1088 (±390)	1174 (±408)	1006 (±370)	1261 (±379)	1069 (±335)	0.12
Birthweight < 1000 g	20	12	14	7	15	
Multiples, *n* (%)	17 (35.4%)	6 (17.1%)	8 (30.8%)	10 (37.0%)	9 (30%)	0.4
BPD, *n* (%)	6 (12.5%)	5 (14.3%)	4 (15.4%)	2 (7.4%)	0 (0.0%)	0.71
IVH ≥ III, *n* (%)	1 (2.1%)	0 (0.0%)	1 (3.8%)	1 (3.7%)	2 (6.7%)	0.43
PVL, *n* (%)	1 (2.1%)	0 (0.0%)	0 (0.0%)	1 (3.7%)	0 (0.0%)	0.6
ROP ≥ 3, *n* (%)	3 (6.3%)	2 (5.7%)	1 (3.8%)	0 (0.0%)	0 (0.0%)	0.84
NEC ≥ 2, *n* (%)	0 (0.0%)	1 (2.9%)	0 (0.0%)	0 (0.0%)	1 (3.3%)	0.53
FIP, *n* (%)	3 (6.3%)	1 (2.9%)	0 (0.0%)	0 (0.0%)	0 (0.0%)	0.23
Any neonatal morbidity; *n* (%)	7 (14.6%)	6 (17.1%)	6 (23.1%)	2 (7.7%)	3 (6.7%)	0.5
Surgery, *n* (%)	5 (10.4%)	5 (14.3%)	2 (7.7%)	2 (7.4%)	4 (13.3%)	0.86

PMA = Postmenstrual Age, BPD = Bronchopulmonary Dysplasia, IVH = Intraventricular Haemorrhage, PVL = Periventricular Leukomalacia, ROP = Retinopathy of Prematurity, NEC = Necrotising Enterocolitis, FIP = Focal Intestinal Perforation; data given as mean (SD = standard deviation); ^a^ PMA 23.7, 24.0, and 27.7 weeks; * BPD, IVH ≥ Grade III, PVL, ROP ≥ Stage 3 or intervention, NEC ≥ Stage 2.

**Table 2:** Discharge characteristics of all cohorts.

	Baseline Cohort	Cohort 1	Cohort 2	Cohort 3	Cohort 4	ANOVA *p* Value
*n*	48	35	26	27	30	
PMA at discharge, weeks (SD)	37.8 (±2.1)	37.5 (±2.9)	37.8 (±4.0)	36.9 (±2.8)	36.4 (±1.9)	0.21
O_2_ at discharge, *n* (%)	6 (12.5%)	5 (14.3%)	7 (26.9%)	3 (11.1%)	1 (3.3%)	0.17
Tube feeding at discharge, *n* (%)	3 (6.3%)	4 (11.4%)	12 (46.2%)	13 (48.1%)	22 (73.3%)	<0.01
Home monitoring, *n* (%)	10 (20.8%)	8 (22.9%)	16 (61.5%)	9 (33.3%)	23 (76.7%)	<0.01
Caffeine therapy at discharge, *n* (%)	0 (0.0%)	0 (0.0%)	0 (0.0%)	1 (3.7%)	11 (36.7%)	<0.01
Discharge with any neonatal morbidity *, *n* (%)	7 (14.6%)	6 (17.1%)	6 (23.1%)	2 (7.4%)	3 (10%)	0.5
PMA at discharge in infants with neonatal morbidities *, weeks (SD)	41.1 (±2.8)	42.0 (±3.6)	41.6 (±6.8)	43.5 (±7.4)	39.6 (±1.8)	0.75
Post hoc analysis including only infants with a PMA of 24.0 to 29.9 weeks						
*n*	23	18	18	13	17	
PMA at discharge, weeks (SD)	38.7 (±1.97)	37.7 (±3.19)	36.9 (±1.67)	38.0 (±3.55)	36.4 ^#^ (±1.92)	0.04

PMA = Postmenstrual Age; data given as mean (SD = standard); * BPD, IVH ≥ Grade III, PVL, ROP ≥ Stage 3 or intervention, NEC ≥ Stage 2; ^#^ Post hoc Dunnett test, significantly different from baseline cohort (*p* < 0.05).

**Table 3:** Readmission characteristics across all cohorts.

	Baseline Cohort	Cohort 1	Cohort 2	Cohort 3	Cohort 4	ANOVA *p* Value
*n*	48	35	26	27	30	
Planned readmission, *n* (%)	6 (12.5%)	6 (17.1%)	9 (34.6%)	7 (25.9%)	14 (46.7%)	0.008
Unplanned readmission, *n* (%)	7 (14.6%)	2 (5.7%)	2(7.7%)	3 (11.1%)	2 (6.7%)	0.75
Reasons unplanned readmission	3 Hernia 2 viral infection 1 gastro-oesophageal reflux 1 Hypothermia	1 Medication error 1 BPD deterioration	1 constipation 1 fussiness	1 Inguinal hernia 1 viral infection 1 constipation	1 inguinal hernia 1 gastro-oesophageal reflux	

BPD = Bronchopulmonary Dysplasia.

**Table 4:** Infants without neonatal morbidities *: patient and discharge characteristics of each cohort.

	Baseline Cohort	Cohort 1	Cohort 2	Cohort 3	Cohort 4	ANOVA *p*-Value
Discharge without any neonatal morbidities * (*n*)	41	29	20	25	27	
PMA at discharge, weeks (SD)	37.2 (±1.4)	36.5 (±1.5)	36.6 (±1.8)	36.4 (±1.5)	36.1 ^#^ (±1.6)	0.036
O_2_ at discharge, *n* (%)	1 (2.4%)	2 (6.9%)	2 (10.0%)	2 (8.0%)	1 (3.7%)	0.76
Tube feeding at discharge, *n* (%)	1 (2.4%)	2 (6.9%)	7 (35.0%)	13 (52.0%)	20 (74.1%)	<0.001
Home monitoring, *n* (%)	4 (9.8%)	5 (17.2%)	10 (50.0%)	8 (32.0%)	20 (74.1%)	<0.001
Caffeine therapy at discharge, *n* (%)	0 (0.0%)	0 (0.0%)	0 (0.0%)	1 (4.0%)	11 (40.7%)	<0.001
Socio-medical follow-up care, *n* (%)	31 (75.6%)	17 (58.6%)	16 (80.0%)	18 (72.0%)	22 (81.5%)	0.32
Unplanned readmission, *n* (%)	4 (9.8%)	0 (0.0%)	1 (5.0%)	3 (12.0%)	2 (7.4%)	0.44
Planned readmissions, *n* (%)	5 (12.2%)	4 (13.7%)	5 (25.0%)	6 (24.0%)	12 (44.4%)	0.23

PMA = Postmenstrual Age, BPD = Bronchopulmonary Dysplasia, IVH = Intraventricular Haemorrhage, PVL = Periventricular Leukomalacia, ROP = Retinopathy of Prematurity, NEC = Necrotising Enterocolitis, data given as mean, SD = standard deviation; * BPD, IVH > Grade III, PVL, ROP > Stage 3 or intervention, NEC > Stage 2; ^#^ Post hoc Dunnett test, significantly different from baseline cohort (*p* < 0.05).

**Table 5:** Parental self-assessment regarding discharge of each cohort (infants without neonatal morbidities *).

	Baseline Cohort	Cohort 1	Cohort 2	Cohort 3	Cohort 4	Chi^2^
*n*	33	27	15	22	23	
Time point of discharge: too early, *n* (%)	2/32 ^#^ (6.3%)	2/27 (7.4%)	0/15 (0.0%)	0/22 (0.0%)	1/23 (4.3%)	
Time point of discharge: right, *n* (%)	25/32 ^#^ (78.1%)	23/27 (85.2%)	12/15 (80.0%)	22/22 (100.0%)	22/23 (95.6%)	0.16
Time point of discharge: too late, *n* (%)	5/32 ^#^ (15.6%)	2/27 (7.4%)	3/15 (20.0%)	0/22 (0.0%)	0/22 ^#^ (0.0%)	
Preparedness score for discharge 4–5/5, *n* (%)	25/33 (75.8%)	22/27 (81.5%)	15/15 (100.0%)	19/22 (86.4%)	22/23 (95.7%)	0.22
Stress level 4–5/5 with feeding, *n* (%)	3/33 (9.4%)	9/27 (33.3%)	5/15 (33.3%)	6/22 (27.3%)	6/22 ^#^ (27.3%)	0.28

* BPD, IVH ≥ Grade III, PVL, ROP ≥ Stage 3 or intervention, NEC ≥ Stage 2; BPD = bronchopulmonary dysplasia, IVH = intraventricular haemorrhage, PVL = periventricular leukomalacia, ROP = retinopathy of prematurity, NEC = necrotising enterocolitis, ^#^ Missing answer on questionnaires.

## Data Availability

The raw data supporting the conclusions of this article will be made available by the authors on request. The data are not publicly available due to ongoing of the study.

## Supplementary Materials

The following supporting information can be downloaded at: https://www.mdpi.com/article/10.3390/children11111316/s1, Supplement S1: Self-Assessment Questionnaire, Supplement S2: Discharge Checklist, Supplement S3: CONSORT Flow Diagram.
