# Supplementary material for: Enhancement of Family-Centred Care Is Associated with a Reduction in Postmenstrual Age at Discharge in Preterm Infants
Source: Children (Basel). 2024 Oct 29;11(11):1316. doi: 10.3390/children11111316 (PMC11593165; doi:10.3390/children11111316)

Supplement S1: self-assessment questionnaire in caregiving skills based on van Veenendaal NR, Auxier JN, van der Schoor SRD, Franck LS, Stelwagen MA, de Groof F, et al. Development and psychometric evaluation of the CO-PARTNER tool for collaboration and parent participation in neonatal care. PLoS One. 2021;16(6):e0252074.

Patient label **Please fill in once a week (on Wednesdays)** Date

	The nurse does this	I do this together with the nurse	I do this alone (without the nurse´s help)	I feel safe to do this at home	This is not applicable
Bath my child/clean my child with a washcloth					
Change my child's diaper					
Feed my child (breast or bottle)					
Change my child's clothing					
Get my child out of the incubator/bed					
Give my child medication					
Weigh my child					
Keeping track of output (urination and defecation) of my child					
Measure the temperature of my child					
Keep track of my child's weight					
Keep track of drinking and my child´s feeds					
Give tube feeding to my child					
	The nurse does this	I do this together	I do this alone (without the	I feel safe to do this at home	This is not applicable

		with the nurse	nurse´s help)		
Look at my child’s monitor and handling accordingly (e.g. stimulating during a bradycardia)					
Regulate the visiting of others for my child					
Participate in the daily rounds with the doctor					
Hold/cuddle my child					
Comfort my child whenever she/he needs it					
Kangaroo care/ skin to skin contact					
Be together with my child, be close with my child (intimate time)					
Be together with my child (be present)					
Soothe my child during a painful procedure (for instance drawing blood)					
Recognize my child’s signals					

I would like to learn/take over in the coming week:

Supplement S2: Checklist before discharge

Discharge Checklist

Dear family,

here is a list of things that need to be done before I can go home. Don't worry, we can do this together. Thank you for preparing everything for me!

**Family**	**Necessary**	**Done**	**Team neonatology**	**Necessary**	**Done**
Equipment at home ready (bed, sleeping bag, diapers, care articles, fever thermometer, breast pump)	x		Hearing test	x	
Pediatrician's name:	x		Pulse oxymetry screening for critical heart disease	x	
Trainings completed:			Vaccination according to vaccination plan	x	
• Safe sleep	x		RSV immunization		
• Tube feeding	x		BPD testing 36 weeks PMA	x	
• Checking and placing a feeding tube			Order home monitor		
• Home monitor			Order oxygen tank		
• Newborn resuscitation			Order feeding tube equipment		
• Prevention of viral infection	x		Ultrasound hip	x	
• Video on newborn care	x		Ultrasound kidney	x	
Oxygen tank arrived at home			Ultrasound head	x	
Feeding tubes arrived at home			Echocardiography		
Take remaining breast milk with you			Blood count (if necessary, adjust iron supplementation)	x	
Infant formula/ breast milk supplement procured	x		Outpatient appointments arranged:<br>- Neonatology<br>- Ophthalmologist<br>- …	x	

Able to administer all medication independently	x		Social service consultation	x	
Pick up medication from pharmacy	x		Socio-medical follow- up program approved by health insurance	x	
Midwife	x		Healthy child check- up documented	x	
			Discharge letter completed and reviewed	x	
			Percentile	x	

Discharge takes place until 12am, we ask parents to be on the ward from 9am on the day of discharge.

Room for questions:

Supplement S3: CONSORT Flow Diagram

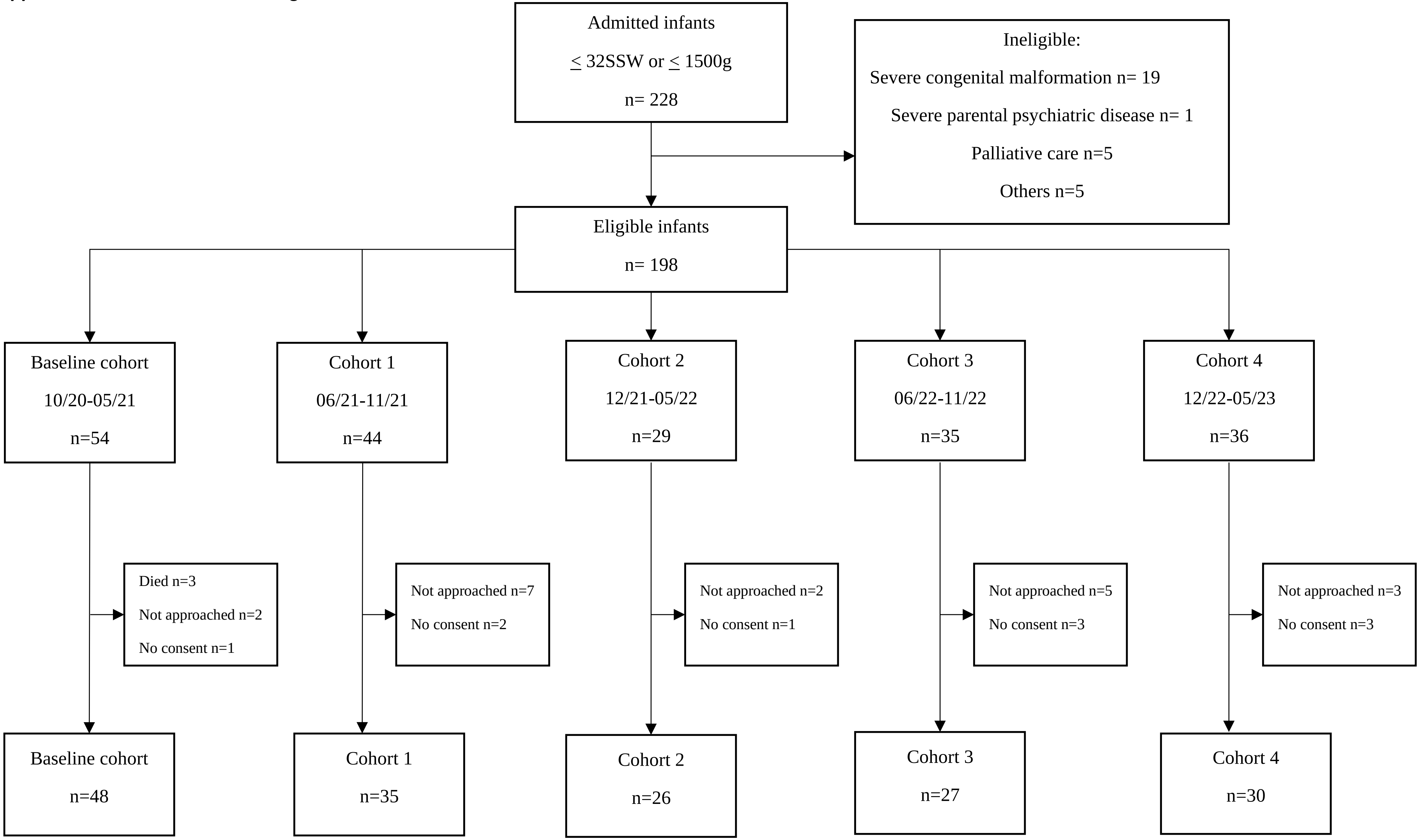

Supplement: Supplementary file 1 [file children-11-01316-s001.zip › children-3256644-supplementary.pdf]
